# Characteristics of Channel Eigenvalues and Mutual Coupling Effects for Holographic Reconfigurable Intelligent Surfaces

**DOI:** 10.3390/s22145297

**Published:** 2022-07-15

**Authors:** Shu Sun, Meixia Tao

**Affiliations:** 1 Department of Electronic Engineering, School of Electronic Information and Electrical Engineering, Shanghai Jiao Tong University, Shanghai 200240, China; mxtao@sjtu.edu.cn; 2 Shanghai Key Laboratory of Digital Media Processing and Transmissions, Shanghai 200240, China

**Keywords:** reconfigurable intelligent surface (RIS), spatial correlation, eigenvalue, spatial degrees of freedom, mutual coupling, holographic communications

## Abstract

As a prospective key technology for the next-generation wireless communications, reconfigurable intelligent surfaces (RISs) have gained tremendous research interest in both the academia and industry in recent years. Only limited knowledge, however, has been obtained about the channel eigenvalue characteristics and spatial degrees of freedom (DoF) of systems containing RISs, especially when mutual coupling (MC) is present between the array elements. In this paper, we focus on the small-scale spatial correlation and eigenvalue properties excluding and including MC effects, for RISs with a quasi-continuous aperture (i.e., holographic RISs). Specifically, asymptotic behaviors of far-field and near-field eigenvalues of the spatial correlation matrix of holographic RISs without MC are first investigated, where the counter-intuitive observation of a lower DoF with more elements is explained by leveraging the power spectrum of the spatial correlation function. Second, a novel metric is proposed to quantify the inter-element correlation or coupling strength in RISs and ordinary antenna arrays. Furthermore, in-depth analysis is performed regarding the MC effects on array gain, effective spatial correlation, and eigenvalue architectures for a variety of element intervals when a holographic RIS works in the radiation and reception mode, respectively. The analysis and numerical results demonstrate that a considerable amount of the eigenvalues of the spatial correlation matrix correspond to evanescent waves that are promising for near-field communication and sensing. More importantly, holographic RISs can potentially reach an array gain conspicuously larger than conventional arrays by exploiting MC, and MC has discrepant impacts on the effective spatial correlation and eigenvalue structures at the transmitter and receiver.

## 1. Introduction

The sixth-generation (6G) communication networks is envisioned to embrace numerous new use cases and challenging requirements [1]. Among the emerging candidate physical-layer technologies for 6G, reconfigurable intelligent surfaces (RISs), sometimes also named large intelligent surfaces [2] and holographic multiple-input multiple-output (MIMO) [3], shows promising foreground in capacity and coverage enhancement, reconfigurable environment construction, intelligent sensing and control, and holographic communications [2,3,4,5,6,7,8,9]. We utilize *holographic RIS* [7,10] herein as an umbrella term for the two-dimensional (2D) architectures with an element spacing equal to or smaller than half a wavelength of the carrier frequency, which can be perceived as an extension of massive MIMO [11] with the ultimate form of (approximately) spatially-continuous electromagnetic (EM) aperture [12]. Holographic RISs can be employed at the base station (BS), user equipment (UE), and/or interacting objects in the propagation medium, and is likely to bring immense advantages in not only spectral efficiency, energy efficiency, and system scalability inherited from massive MIMO [13,14,15], but also the manipulation of EM waves via anomalous reflection, refraction, polarization transformation, and so on [3], reminiscent of metasurfaces in the optical regime [16,17]. In order to unleash the full potentials of holographic RISs, it is necessary to understand its fundamental properties, such as channel eigenvalues and spatial degrees of freedom (DoF).

### 1.1. Related Work

The use of dense antenna arrays for wireless communications was in fact explored over two decades ago (e.g., [18,19,20]), and has resurged recently as a promising 6G technology [2,3,12]. Due to the limited element spacing and 2D (as opposed to one-dimensional) structure of a dense array, the spatial correlation between array elements is not always non-zero even under isotropic scattering [12,21,22]. Among the early work involving antenna spatial correlation, the authors in [23] have considered spatial correlation among multi-element antennas, and derived upper and lower capacity bounds taking into account antenna correlation. Fading correlation has also been studied in [24] to examine the capacity growth with respect to the number of antenna elements. The impact of antenna correlation on capacity has been examined for diverse signal inputs and correlation architectures in [25]. Nevertheless, the antenna element spacing is of half-wavelength or larger in [23,24,25]. The capacity of spatially dense multiple antenna systems has been pioneered in [18], which showed that the capacity of such a system approaches a finite limit. Spatially dense MIMO arrays have also been studied in [19], where the array-gain normalized capacity has been analyzed and the performance of small wavelength-like MIMO arrays has been shown to be similar to that of arrays with larger apertures. The asymptotic capacity associated with antenna arrays of fixed length has been analyzed in [20] for uniform linear antenna arrays, revealing that the asymptotic mutual information converges almost surely as the number of antenna elements approaches infinity due to the convergence of the eigenvalues. The aforementioned work, however, focused mainly on the capacity and did not explicitly consider the spatial DoF that the arrays can offer.

The spatial DoF for holographic RISs in line-of-sight (LoS) environments have been investigated in [26], which has revealed that the DoF can be larger than one even in strong LoS channel conditions, favorable for spatial multiplexing. In [2,12], the asymptotic spatial DoF for sufficiently dense and large holographic RISs have been derived from the perspectives of channel capacity and Fourier plane-wave series expansion, respectively. The achievable DoF for more common cases with finite element spacing and aperture areas has been investigated in [22], where it has been discovered that the spatial DoF decreases as the number of elements grows which seems counter-intuitive, but the underlying causes have not been identified.

Due to the close proximity of neighboring elements in a holographic RIS, mutual coupling (MC) naturally arises. Broadly speaking, MC refers to EM interaction among the array elements and can occur because of three mechanisms: direct space coupling between array elements, indirect coupling caused by near-by scatterers, and coupling through feed network [27,28]. In this paper, we mainly refer to MC stemming from the first mechanism. MC between array elements can be characterized by a conventional multi-port circuit model, such as an impedance matrix, an admittance matrix, or a scattering matrix. Theoretically, the type of matrix used to represent the array network is not important since matrix transforms can be applied to change the type of matrix representation. There is abundant early research work on MC in antenna arrays (e.g., [28,29,30,31,32,33,34,35,36] and references therein), which considered linear arrays only or did not fix the array aperture while varying the element spacing. Aiming at exploring the physical characteristics of the emerging holographic RISs or similar structures for 6G communications, the authors in [37] have proposed an LoS communication model incorporating MC in the form of mutual impedance, and studied the associated optimal beamforming strategies. The model has then been extended in [38] to account for superdirectivity and MC effects as well as near-field propagation. An end-to-end MC-aware communication model based on mutual impedances has been propounded in [39], which is also EM-compliant and unit cell aware. In [40], a circuit-based MIMO channel model considering antenna MC, size-related antenna equivalent circuit, and channel small-scale fading has been proposed, and statistical properties including temporal autocorrelation function and spatial cross-correlation function, along with the effects of MC and size-related antenna equivalent circuit on them have been revealed. The authors in [41] have derived a beamforming vector of superdirective arrays based on a coupling matrix-enabled method, and proposed an approach to obtain the coupling matrix via spherical wave expansion.

### 1.2. Contributions

Despite the aforementioned extensive research work, study on small-scale spatial characteristics, the associated MC effects, and the mechanism behind some unique phenomena for holographic RISs are still in the infancy. In this paper, therefore, we carry out thorough investigation on the aspects above. Specifically, the small-scale spatial correlation, eigenvalue behavior, and spatial DoF for holographic RISs excluding and including MC are explored. The major novelty and contributions of this article lie in the following aspects:

- First, leveraging the block-Toeplitz with Toeplitz block (BTTB) matrix theory, we relate the eigenvalues of the spatial correlation matrix of the holographic RIS to the power spectrum of the spatial correlation function, and explain the counter-intuitive phenomenon of seemingly lower spatial DoF with growing numbers of elements in a holographic RIS observed in our prior work [22], which has not been addressed in the literature to our best knowledge. This analysis also helps with distinguishing the spatial DoF corresponding to the far field and near field of a holographic RIS.
- Second, we incorporate MC into the array response and spatial correlation matrix of the holographic RIS considering realistic element sizes, and demonstrate the potential of holographic RISs to reach an extraordinary array gain that is significantly higher than conventional antenna arrays with concrete examples. The results indicate that, different from the common belief that MC is always deleterious and should be avoided or compensated for, MC can be beneficial in boosting the array gain of holographic RISs even without sophisticated manipulation of excitation coefficients for the array elements.
- Furthermore, in-depth analysis and comparisons are performed regarding the MC effects on spatial correlation and the corresponding eigenvalue distributions for holographic RISs working in the transmitting (Tx) and receiving (Rx) modes, respectively, and with various element intervals as well as source and load impedance values. A metric named *inter-element correlation/coupling strength indicator (ICSI)* is proposed to measure the amount of inter-element correlation/coupling within an array. Results show that the effects of MC are quite discrepant for Tx and Rx arrays, and are also dependent upon element spacing, source and load impedance, among other factors, necessitating comprehensive design and implementation considerations.

Isotropic scattering is considered in this paper, since it is a typical type of environment involved in an enormous amount of theoretical research work, and also encountered by low-frequency bands (e.g., sub-1 GHz) which are still crucial even in 6G to guarantee wide coverage and high reliability [42], and the corresponding results can serve as theoretical upper bounds for non-isotropic scattering scenarios. The contributions in this paper unveil some fundamental channel eigenvalue features and practical spatial DoF of holographic RISs, and provide valuable hints on channel estimation and beamforming strategies for holographic RISs [38,41,43].

### 1.3. Article Outline and Notation

The remainder of this paper is organized as follows: in Section 2, we describe the system model, and formulate array responses and spatial correlation excluding and including MC for holographic RISs. Analyses of asymptotic eigenvalue distributions of the spatial correlation matrix without and with MC are provided in Section 3 and Section 4, respectively. Conclusions are drawn in Section 5.

The following notations will be utilized throughout the paper: A for matrix, a for column vector, ${\left[A\right]}_{i,j}$ for the (i,j)th entry of A; $A^{T}$, $A^{*}$, and $A^{H}$ for the transpose, conjugate, and Hermitian of A, respectively; det(A) for determinant of the square matrix A, tr(A) for the trace of A, $I_{N}$ for the N×N identity matrix, while ⌈a⌉ and ⌊a⌋ for the ceiling and floor of the scalar *a*, respectively.

## 2. System Model

**Figure 1:**
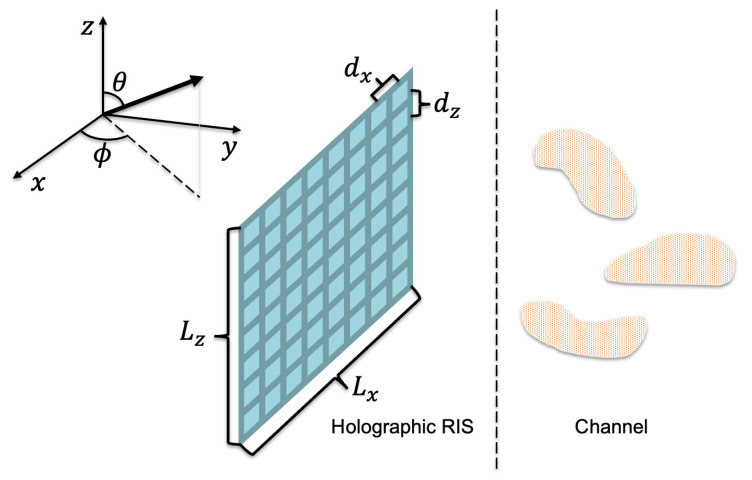
System model and orientation of the holographic reconfigurable intelligent surface (RIS) with respect to the associated coordinate system. The horizontal and vertical lengths of the holographic RIS are $L_{x}$ and $L_{z}$, with element spacing of $d_{x}$ and $d_{z}$, respectively. The azimuth and zenith angles are denoted by ϕ and θ, respectively, and the three irregular blocks in the channel represent random scatterers.

We consider a holographic RIS in a wireless communication system where the holographic RIS can be employed at the BS, UE, and/or interacting objects that can radiate, receive, reflect, or refract wireless signals, as illustrated in Figure 1. The horizontal and vertical lengths of the holographic RIS are $L_{x}$ and $L_{z}$, with element spacing of $d_{x}$ and $d_{z}$, respectively. Each element in the holographic RIS is modeled as a cylindrical thin wire of perfectly conducting material, and is connected to a tunable load, where the load can be a positive-intrinsic-negative diode whose inductance and capacitance are adaptable to reconfigure the response of each element [39].

The signal sent from or impinging on the holographic RIS is generally composed of a superposition of multipath components which can be regarded as a continuum of plane waves, hence the channel capturing small-scale fading can be expressed as [44]

(1) $$h=\int_{0}^{\pi }\int_{0}^{\pi }\tilde{s}\left(\phi ,\theta \right)a\left(\phi ,\theta \right)d\phi d\theta$$

where $\tilde{s}\left(\phi ,\theta \right)$ denotes the angular distribution function that contains the channel gain and phase shift corresponding to the direction (ϕ,θ) with ϕ and θ representing the azimuth and zenith angles, respectively, while a(ϕ,θ) is the array response vector. The correlation function is given by

(2) $$R=E\left\{hh^{H}\right\}=\int_{0}^{\pi }\int_{0}^{\pi }s\left(\phi ,\theta \right)a\left(\phi ,\theta \right)a^{H}\left(\phi ,\theta \right)d\phi d\theta$$

where s(ϕ,θ) denotes the normalized spatial scattering function satisfying

(3) $$E\left\{\tilde{s}\left(\phi ,\theta \right){\tilde{s}}^{*}\left(\phi^{\prime },\theta^{\prime }\right)\right\}=s\left(\phi ,\theta \right)\delta \left(\phi -\phi^{\prime }\right)\delta \left(\theta -\theta^{\prime }\right)$$

and $\int_{0}^{\pi }\int_{0}^{\pi }s\left(\phi ,\theta \right)d\phi d\theta =1$. Physically, the presence of spatial correlation means that the signal strengths at different elements do not vary independently, but may rise or fade simultaneously.

### 2.1. Array Response and Spatial Correlation Excluding MC

For an array with *N* elements, where each element has the same pattern function of p(ϕ,θ) (Strictly speaking, if MC exists, the central elements and the ones near the array edges may not maintain the same element pattern when embedded in an array, even if their isolated element patterns are identical [27]. Nevertheless, it is possible to compensate for the pattern distortion via predetermined illumination, and here we assume the same embedded element pattern for all the elements in an array. The element pattern variation owing to MC is another topic and is deferred to future work), the far-field radiation pattern of the array is expressed as

(4) $$f\left(\phi ,\theta \right)=\sum_{n=1}^{N}w_{n}p\left(\phi ,\theta \right)e^{j\kappa \hat{d}\cdot d_{n}}$$

where $w_{n}$ denotes the complex excitation coefficient proportional to the current on the *n*-th element, κ=2π/λ is the wavenumber with λ being the carrier wavelength, $\hat{d}$ represents the unit vector of the far-field direction (ϕ,θ) in the spherical coordinate system, and $d_{n}$ is the position vector of the *n*-th element. (4) holds if there is no MC among the array elements, which is usually the case when the spacing between adjacent elements is sufficiently large (e.g., more than a couple of wavelengths). Accordingly, the conventional MC-unaware array response vector is defined as

(5) $$a_{0}={\left[e^{j\kappa \hat{d}\cdot d_{1}},\ldots ,e^{j\kappa \hat{d}\cdot d_{n}},\ldots ,e^{j\kappa \hat{d}\cdot d_{N}}\right]}^{T}.$$

Then, the correlation matrix $R_{0}$ excluding the MC effects is given by

(6) $$R_{0}=\int_{0}^{\pi }\int_{0}^{\pi }s\left(\phi ,\theta \right)a_{0}\left(\phi ,\theta \right)a_{0}^{H}\left(\phi ,\theta \right)d\phi d\theta .$$

The concrete expression and properties of $R_{0}$ will be provided in Section 3 to investigate the characteristics of its eigenvalues.

### 2.2. Array Response and Spatial Correlation Including MC

In a holographic RIS, the elements are densely arranged so that MC is usually non-negligible. The element pattern considering MC can be formulated as [32]

(7) $$\tilde{p}\left(\phi ,\theta \right)=p\left(\phi ,\theta \right)\sum_{m=1}^{N}c_{mn}e^{j\kappa \hat{d}\cdot d_{m}}$$

where $c_{mn}$ represents the coupling coefficient between the *m*-th and the *n*-th elements. Namely, the pattern of the *n*-th element can be modeled as the original element pattern p(ϕ,θ) times a weighted sum of impact from all the other elements. Thus, the radiation pattern of the array incorporating MC follows

(8) $$\tilde{f}\left(\phi ,\theta \right)=\sum_{n=1}^{N}\sum_{m=1}^{N}w_{n}p\left(\phi ,\theta \right)c_{mn}e^{j\kappa \hat{d}\cdot d_{m}}.$$

The coupling matrix collecting the MC coefficients is written as

(9) $$C=\left[\begin{array}{cccc} c_{11} & c_{12} & \cdots & c_{1N}\\ c_{21} & c_{22} & \cdots & c_{2N}\\ \vdots & \vdots & \ddots & \vdots \\ c_{N1} & c_{N2} & \cdots & c_{NN} \end{array}\right].$$

When there is no MC among the elements, C reverts to an identity matrix. It can be derived from (5), (7) and (9) that the effective array response vector a involving MC is given by

(10) $$a=C^{T}a_{0}$$

in which $a_{0}$ stands for the original MC-unaware array response vector in (5).

Now, let us look at a crucial metric relevant to the array response—array gain, which is defined herein as the increase in radiation power of an array compared with that of a single element under the same total excitation power. It is well known in the antenna literature [45,46,47] that the array gain of an array with closely-spaced elements can grow with the square of the number of elements, and rigorous proof for a linear array was provided in [46] with optimal beamforming in the end-fire direction. For an arbitrary pointing direction (ϕ,θ), the array gain $G_{\mathrm{array}}$ incorporating MC can be formulated based on (8) as

(11) $$\begin{array}{cc} G_{\mathrm{array}}\left(\phi ,\theta \right) & =\frac{|\sum_{n=1}^{N}\sum_{m=1}^{N}w_{n}p\left(\phi ,\theta \right)c_{mn}e^{j\kappa \hat{d}\cdot d_{m}}{|}^{2}}{|w_{0}p{\left(\phi ,\theta \right)|}^{2}}\\ \; & =\frac{|\sum_{n=1}^{N}\sum_{m=1}^{N}w_{n}c_{mn}e^{j\kappa \hat{d}\cdot d_{m}}{|}^{2}}{|w_{0}{|}^{2}}\\ \; & =\frac{|a^{T}w{|}^{2}}{|w_{0}{|}^{2}}{,\;s.t.\;||w||}_{2}=\left|w_{0}\right| \end{array}$$

where $w_{0}$ stands for the complex excitation coefficient for a single element such that $|w_{0}{|}^{2}$ represents the total excitation power, a is given in (10), and w denotes the beamforming column vector consisting of the complex excitation coefficients $w_{n}$ for an array. Note that w is inherently a function of the pointing direction (ϕ,θ) due to a. Consequently, the optimal beamforming vector maximizing $G_{\mathrm{array}}\left(\phi ,\theta \right)$ is

(12) $$\begin{array}{c} w_{\mathrm{opt}}=\zeta a^{*}=\zeta C^{H}a_{0}^{*} \end{array}$$

in which ζ is a normalization factor equal to $\frac{|w_{0}|}{\left|\right|C^{H}a_{0}^{*}{\left|\right|}_{2}}$ to satisfy the power constraint. Plugging (12) into (11) produces the maximum array gain at the direction (ϕ,θ)

(13) $$\begin{array}{c} G_{\mathrm{array}}{\left(\phi ,\theta \right)}_{\max}=|a_{0}^{T}CC^{H}a_{0}^{*}|. \end{array}$$

It is straightforward to observe from (13) that, when excluding MC, i.e., when C is an identity matrix, the maximum array gain is $|a_{0}^{T}a_{0}^{*}|=N$ for an arbitrary direction. When taking MC into account, the maximum array gain becomes $\left|a_{0}^{T}CC^{H}a_{0}^{*}\right|$ which is usually larger than *N* as will be shown later by simulations, and varies with pointing angles. The array gain in (13) of a holographic RIS is highly promising, since it indicates that, compared with a traditional array with *N* elements, the received signal-to-noise ratio (SNR) can be enhanced by up to $\frac{\left|a_{0}^{T}CC^{H}a_{0}^{*}\right|}{N}$ fold for a fixed transmit power using a holographic RIS with the same number of elements; or, equivalently, the transmit power can be scaled down by up to a factor of $\frac{N}{\left|a_{0}^{T}CC^{H}a_{0}^{*}\right|}$ without compromising the received SNR. The disadvantages of the proposed method in (12) are that the coupling matrix C needs to be known (e.g., through rigorous theoretical analysis or measurements) before conducting the beamforming, and that the achievable array gain in (13) might be smaller than *N* in some corner cases (as will be shown later via simulations) depending on the properties of the coupling matrix C and target pointing angles.

Based upon the array response vector incorporating MC in (10), the effective spatial correlation matrix R in (2) is expanded as

(14) $$\begin{array}{cc} R & =E\left\{hh^{H}\right\}\\ \; & =\int_{0}^{\pi }\int_{0}^{\pi }s\left(\phi ,\theta \right)C^{T}a_{0}a_{0}^{H}\left(\phi ,\theta \right)C^{*}d\phi d\theta \\ \; & =C^{T}\left(\int_{0}^{\pi }\int_{0}^{\pi }s\left(\phi ,\theta \right)a_{0}a_{0}^{H}\left(\phi ,\theta \right)d\phi d\theta \right)C^{*}\\ \; & =C^{T}R_{0}C^{*} \end{array}$$

which implies that each entry in the effective spatial correlation matrix R is determined by the collective effects of all the entries in the original spatial correlation matrix $R_{0}$ weighted by the relevant entries in the coupling matrix C. The theoretical analysis in (12)–(14) will be applied in Section 4 to study the performance of the proposed beamforming approach and the influence of MC on the effective spatial correlation of holographic RISs.

## 3. Eigenvalue Distributions Without MC

Denote the number of elements in a holographic RIS along the *x* and *z* directions as $N_{x}$ and $N_{z}$, respectively, i.e., the total number of elements $N=N_{x}N_{z}$. For the isotropic scattering environment, the spatial scattering function in (2) is [22]

(15) $$s\left(\phi ,\theta \right)=\frac{\sin\theta }{2\pi },\;\phi \in \left[0,\pi \right],\theta \in \left[0,\pi \right].$$

**Figure 2:**
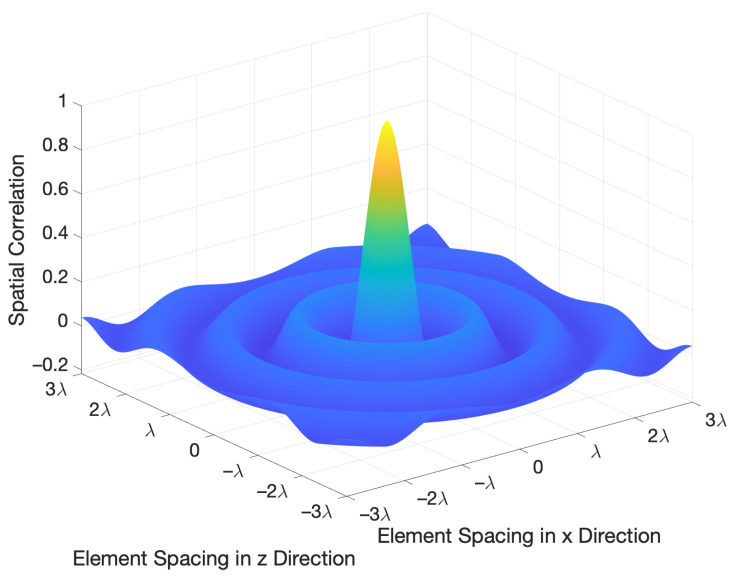
Spatial correlation among the holographic RIS elements under isotropic scattering, where λ denotes the carrier wavelength. Note that the element spacing in *x* and *z* directions are expressed in terms of the wavelength λ, which is directly labeled on the abscissa and ordinate, while the spatial correlation is dimensionless.

Substituting (15) into (6) in Section 2.1 yields the spatial correlation matrix $R_{0}$of the holographic RIS under isotropic scattering. As proven in [21,22], $R_{0}$ can be characterized by a sinc function as follows:

(16) $$\begin{array}{c} {\left[R_{0}\right]}_{n_{1},n_{2}}=\sin c\left(\frac{2||d_{n_{1}}-d_{n_{2}}{\left|\right|}_{2}}{\lambda }\right),\;n_{1},n_{2}=1,\ldots ,N \end{array}$$

where $\sin c\left(x\right)≜\frac{\sin(\pi x)}{\pi x}$ is the sinc function, $d_{n_{1}}$ and $d_{n_{2}}$ denote the coordinates of the $n_{1}$-th and $n_{2}$-th elements in the holographic RIS, respectively. The behavior of small-scale spatial correlation is depicted in Figure 2 for element spacing up to three times the wavelength λ. It is observed from (16) and Figure 2 that the spatial correlation is minimal only for some element spacing, instead of between any two elements, thus the classical independent and identically distributed (i.i.d.) Rayleigh fading model is not applicable in such a system [12,21,22].

**Figure 3:**
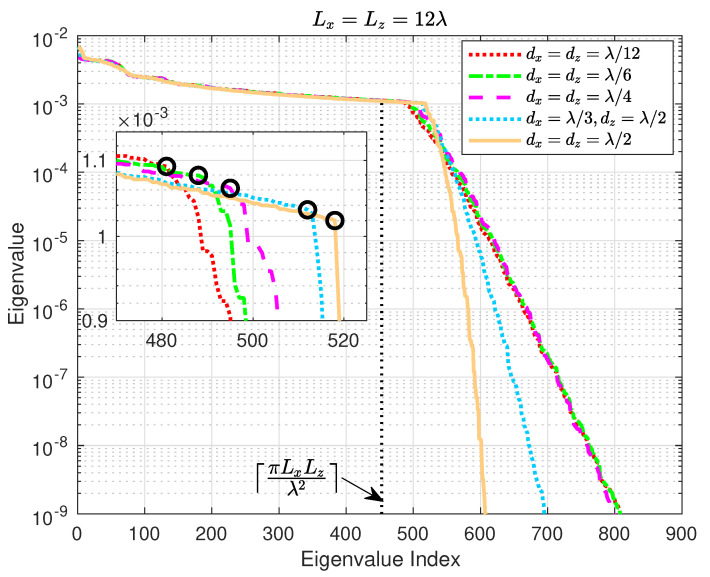
Eigenvalue versus eigenvalue index of $R_{0}/N$ in non-increasing order for various element spacing $d_{x}$ and $d_{z}$ with $L_{x}=L_{z}=12\lambda$. In addition, the asymptotic spatial degrees of freedom (DoF) $\left\lceil \frac{\pi L_{x}L_{z}}{\lambda^{2}}\right\rceil$ derived in [48] are depicted for $\min(L_{x},L_{z})/\lambda \to \infty$. Note that both the eigenvalue and its index are dimensionless. Each dark circle in the inset represents the inflection point where the eigenvalues start to drop rapidly.

Figure 3 portrays the eigenvalues of $R_{0}/N$ in non-increasing order for various *N*, or equivalently various element spacing, with $L_{x}=L_{z}=12\lambda$. In addition, the dotted vertical line represents the asymptotic spatial DoF $\left\lceil \frac{\pi L_{x}L_{z}}{\lambda^{2}}\right\rceil$ for $\min(L_{x},L_{z})/\lambda \to \infty$[48,49]. A few key remarks can be drawn from Figure 3: First, while the popular i.i.d. Rayleigh fading channel has almost identical eigenvalues whose amount equals the number of antenna elements deployed, the correlated channel herein has uneven and fewer dominant eigenvalues (those larger than the ones highlighted by the dark circles in the inset of Figure 3) and smaller rank. Second, the accuracy of the spatial DoF $\left\lceil \frac{\pi L_{x}L_{z}}{\lambda^{2}}\right\rceil$ increases with the element density. More importantly, the number of dominant eigenvalues, highlighted by the dark circles in the inset of Figure 3, declines as the total number of elements *N* increases, which seems counter-intuitive. Therefore, in the following subsections, we will dig into the underlying causes of the aforementioned uncommon phenomenon of seemingly reduced spatial DoF with an increased number of elements in a holographic RIS.

### 3.1. Relationship between Eigenvalues and Power Spectrum

Note that the spatial correlation matrix $R_{0}$ of the 2D holographic RIS in (16) can be formulated as

(17) $$R_{0}=\left[\begin{array}{ccccc} B_{0} & B_{1} & B_{2} & \cdots & B_{N_{z}-1}\\ B_{-1} & B_{0} & B_{1} & \cdots & B_{N_{z}-2}\\ B_{-2} & B_{-1} & B_{0} & \cdots & B_{N_{z}-3}\\ \vdots & \vdots & \vdots & \ddots & \vdots \\ B_{1-N_{z}} & B_{2-N_{z}} & B_{3-N_{z}} & \cdots & B_{0} \end{array}\right]$$

where each block $B_{m}\in C^{N_{x}\times N_{x}}$, $|m|<N_{z}$, is a symmetric Toeplitz matrix by itself, and $B_{-m}=B_{m}^{T}$, i.e., the entire matrix $R_{0}$ is symmetric as well. Moreover, the matrix blocks $B_{m}$’s along each diagonal of $R_{0}$ are identical. Therefore, the spatial correlation matrix $R_{0}$ has a symmetric BTTB structure under isotropic scattering, thus we resort to the relevant theory of the asymptotic distribution of eigenvalues of BTTB forms to investigate the properties of the eigenvalues of $R_{0}/N$.

Denote the (u,v)th entry of $B_{m}$ by $b_{l,m}=b_{u-v,m}$, $u,v=1,\ldots ,N_{x}$, and define the function

(18) $$g\left(\Delta_{x},\Delta_{z}\right)=\sin c\left(\frac{2\sqrt{\Delta_{x}^{2}+\Delta_{z}^{2}}}{\lambda }\right)$$

where $\Delta_{x}$ and $\Delta_{z}$ are the spacing along the *x*- and *z*-axes, respectively, between a pair of spatial points of interest in the xoz plane. $\left\{b_{l,m}\right\}$ can thus be regarded as a finite truncated bi-sequence generated from $g(\Delta_{x},\Delta_{z})$, and more precisely,

(19) $$b_{l,m}=\sin c\left(\frac{2\sqrt{{\left(\frac{lL_{x}}{N_{x}}\right)}^{2}+{\left(\frac{mL_{z}}{N_{z}}\right)}^{2}}}{\lambda }\right).$$

Let $G(\omega_{x},\omega_{z})$ be the 2D Fourier transform of $\left\{b_{l,m}\right\}$ given by

(20) $$\begin{array}{cc} \; & G\left(\omega_{x},\omega_{z}\right)=\frac{1}{N}\sum_{l=-(N_{x}-1)}^{N_{x}-1}\sum_{m=-(N_{z}-1)}^{N_{z}-1}b_{l,m}e^{-j(l\omega_{x}+m\omega_{z})},\\ \; & \omega_{x}=-\frac{(N_{x}-1)\pi }{N_{x}},-\frac{(N_{x}-3)\pi }{N_{x}},\ldots ,\frac{(N_{x}-1)\pi }{N_{x}},\\ \; & \omega_{z}=-\frac{(N_{z}-1)\pi }{N_{z}},-\frac{(N_{z}-3)\pi }{N_{z}},\ldots ,\frac{(N_{z}-1)\pi }{N_{z}}. \end{array}$$

Since the double-index sequence $\left\{b_{l,m}\right\}$ consists of spatial samples of the continuous sinc function $g(\Delta_{x},\Delta_{z})$ in (18), $G(\omega_{x},\omega_{z})$ in (20) can be looked upon as the power spectrum of the discretized and truncated spatial correlation function. According to the properties of BTTB matrices, the eigenvalues of $R_{0}/N$ behave asymptotically the same as the spectral sampling points of $G(\omega_{x},\omega_{z})$ as N→∞, if the double-index sequence formed by $b_{l,m}$ is absolutely summable [50,51], i.e.,

(21) $$\lim_{N_{x},N_{z}\to \infty }\sum_{l=-(N_{x}-1)}^{N_{x}-1}\sum_{m=-(N_{z}-1)}^{N_{z}-1}\left|b_{l,m}\right|\leq \mathrm{Constant}<\infty .$$

The condition in (21) is indeed satisfied as shown by (22),

(22) $$\begin{array}{cc} \; & \lim_{N_{x},N_{z}\to \infty }\sum_{l=-(N_{x}-1)}^{N_{x}-1}\sum_{m=-(N_{z}-1)}^{N_{z}-1}\left|b_{l,m}\right|\\ = & \lim_{N_{x},N_{z}\to \infty }\sum_{l=-(N_{x}-1)}^{N_{x}-1}\sum_{m=-(N_{z}-1)}^{N_{z}-1}\left|\sin c\left(\frac{2\sqrt{{\left(\frac{lL_{x}}{N_{x}}\right)}^{2}+{\left(\frac{mL_{z}}{N_{z}}\right)}^{2}}}{\lambda }\right)\right|\\ = & \int_{-1}^{1}\int_{-1}^{1}\left|\sin c\left(\frac{2\sqrt{{\left(\varpi L_{x}\right)}^{2}+{\left(\varsigma L_{z}\right)}^{2}}}{\lambda }\right)\right|d\varpi d\varsigma \\ \leq & \int_{-1}^{1}\int_{-1}^{1}1d\varpi d\varsigma =4<\infty \end{array}$$

hence the eigenvalues of $R_{0}/N$ and the spectral sampling points of $G(\omega_{x},\omega_{z})$ in (20) are asymptotically equally distributed. Consequently, insights on the eigenvalues of $R_{0}/N$ can be drawn via the investigation of $G(\omega_{x},\omega_{z})$. In what follows, we explore how the power spectrum $G(\omega_{x},\omega_{z})$ changes with the element spacing of the holographic RIS, or equivalently, the spatial sampling frequency, in order to explain the unconventional observation of seemingly lower spatial DoF with growing numbers of elements in a holographic RIS as manifest in Figure 3.

### 3.2. Analysis on Eigenvalues via Power Spectrum

When the element spacing in a holographic RIS is $\eta_{x}\lambda$ and $\eta_{z}\lambda$ ($\eta_{x},\eta_{z}>0$) along the *x* and *z* directions, respectively, and $L_{x}=\beta_{x}\lambda$, $L_{z}=\beta_{z}\lambda$ ($\beta_{x},\beta_{z}>0$), we obtain $N_{x}=\beta_{x}/\eta_{x}+1$, $N_{z}=\beta_{z}/\eta_{z}+1$. The power spectrum $G(\omega_{x},\omega_{z})$ in (20) can be recast as (23),

(23) $$\begin{array}{cc} \; & G\left(\kappa_{x},\kappa_{z}\right)=\frac{1}{N}\sum_{l=-\frac{\beta_{x}}{\eta_{x}}}^{\frac{\beta_{x}}{\eta_{x}}}\sum_{m=-\frac{\beta_{z}}{\eta_{z}}}^{\frac{\beta_{z}}{\eta_{z}}}\sin c\left(2\sqrt{{\left(\frac{l\eta_{x}\left(N_{x}-1\right)}{N_{x}}\right)}^{2}+{\left(\frac{m\eta_{z}\left(N_{z}-1\right)}{N_{z}}\right)}^{2}}\right)e^{-j\lambda (l\eta_{x}\kappa_{x}+m\eta_{z}\kappa_{z})},\\ \; & \kappa_{x}=-\frac{(N_{x}-1)\kappa }{2N_{x}\eta_{x}},-\frac{(N_{x}-3)\kappa }{2N_{x}\eta_{x}},\ldots ,\frac{(N_{x}-1)\kappa }{2N_{x}\eta_{x}},\\ \; & \kappa_{z}=-\frac{(N_{z}-1)\kappa }{2N_{z}\eta_{z}},-\frac{(N_{z}-3)\kappa }{2N_{z}\eta_{z}},\ldots ,\frac{(N_{z}-1)\kappa }{2N_{z}\eta_{z}} \end{array}$$

in which κ=2π/λ denotes the wavenumber; $\kappa_{x}$ and $\kappa_{z}$ represent the wavenumber along the *x* and *z* directions, respectively. The wavenumber along the positive *y* direction in Figure 1 is defined as

(24) $$\varkappa \left(\kappa_{x},\kappa_{z}\right)=\sqrt{\kappa^{2}-\left(\kappa_{x}^{2}+\kappa_{z}^{2}\right)}$$

and the wave vector is

(25) $$\kappa =\kappa_{x}\hat{x}+\varkappa \left(\kappa_{x},\kappa_{z}\right)\hat{y}+\kappa_{z}\hat{z}$$

where $\hat{x}$, $\hat{y}$, and $\hat{z}$ denote the unit vector along the *x*-, *y*-, and *z*-axis, respectively. It is noteworthy that a real-valued $\varkappa (\kappa_{x},\kappa_{z})$ corresponds to a wave propagating along the *y*-direction, while an imaginary-valued $\varkappa (\kappa_{x},\kappa_{z})$ indicates an evanescent wave that usually exists around the surface of an object and decays exponentially in space [12,17].

**Figure 4:**
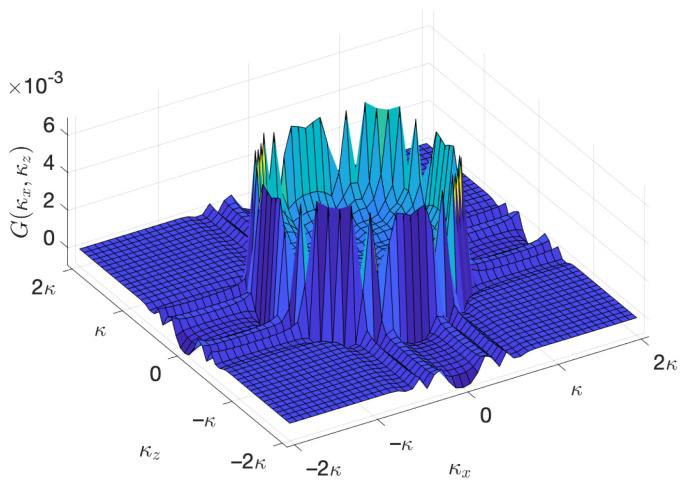
Fourier transform $G(\kappa_{x},\kappa_{z})$ in (23) of the truncated and discretized spatial correlation function $g(\Delta_{x},\Delta_{z})$ in (18) with $L_{x}=L_{z}=12\lambda$ and $d_{x}=d_{z}=\lambda /3$. Note that $\kappa_{x}$ and $\kappa_{z}$ are expressed in terms of the wavenumber κ, which is directly labeled on the abscissa and ordinate, while $G(\kappa_{x},\kappa_{z})$ is dimensionless since $g(\Delta_{x},\Delta_{z})$ is dimensionless.

The formulation in (23) allows us to study the power spectrum $G(\kappa_{x},\kappa_{z})$ as a function of the wavenumbers $\kappa_{x}$ and $\kappa_{z}$, so as to gain more insight on the influence of the spatial sampling frequency on $G(\kappa_{x},\kappa_{z})$, and, equivalently, on the eigenvalues of the normalized spatial correlation matrix $R_{0}/N$, thanks to the time-frequency and space-wavenumber duality [48]. For a continuous and infinitely large holographic RIS aperture, $G(\kappa_{x},\kappa_{z})$ in (23) approaches the following distribution [52]:

(26) $$\begin{array}{c} G^{\left(\infty \right)}\left(\kappa_{x},\kappa_{z}\right)=\frac{\Pi \left(\frac{\sqrt{\kappa_{x}^{2}+\kappa_{z}^{2}}}{2\kappa }\right)}{\frac{\kappa }{2\pi }\sqrt{\kappa^{2}-\left(\kappa_{x}^{2}+\kappa_{z}^{2}\right)}} \end{array}$$

where Π(·) is the rectangle function. As implied by (26), $G^{\left(\infty \right)}\left(\kappa_{x},\kappa_{z}\right)$ assumes a bowl-like shape for a given κ, which increases monotonically with $\sqrt{\kappa_{x}^{2}+\kappa_{z}^{2}}$ inside the region

(27) $$D\left(\kappa \right)=\left\{\left(\kappa_{x},\kappa_{z}\right)\in R^{2}:\kappa_{x}^{2}+\kappa_{z}^{2}\leq \kappa^{2}\right\}$$

achieving the maximum value when $\sqrt{\kappa_{x}^{2}+\kappa_{z}^{2}}=\kappa$, and then transits abruptly to zero outside D(κ). In practical implementations, however, a holographic RIS often has a finite aperture size and is composed of discrete elements, which is equivalent to spatially*truncating* and *sampling* an originally infinite and continuous holographic RIS aperture. Truncating can be thought of as applying a windowing function to an originally infinitely-long signal in the spatial domain, which will cause the signal to appear outside D(κ) in the wavenumber domain after performing the Fourier transform, as displayed in Figure 4. Regarding spatial sampling, it is known from time-frequency-domain signal processing that a finer sampling granularity in the time domain yields a higher resolution in the (traditional) frequency domain. Analogously, for a holographic RIS, a smaller spatial sampling interval, which entails a smaller element spacing, gives rise to a higher resolution in the spatial frequency (i.e., wavenumber) domain. Accordingly, taking the *x* direction as an example, the highest absolute value of the resolvable wavenumber $\kappa_{x}$, namely $\frac{(N_{x}-1)\kappa }{2N_{x}\eta_{x}}$, is inversely proportional to the element spacing $\eta_{x}$ in (23). Specifically, if $N_{x}\gg 1$, for the most common element spacing of half a wavelength (i.e., $\eta_{x}=1/2$), the highest resolvable wavenumber is κ as expected, and it increases to $\frac{\kappa }{2\eta_{x}}$ as $\eta_{x}$ decreases.

**Figure 5:**
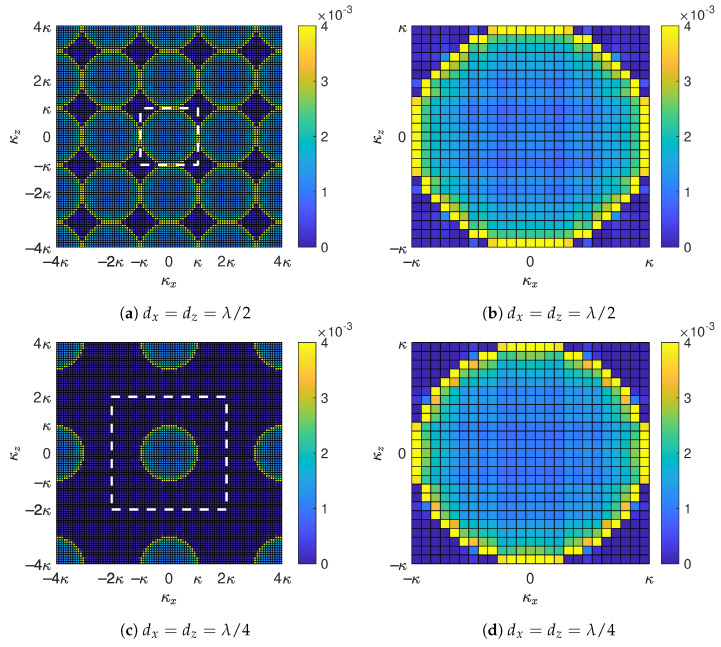

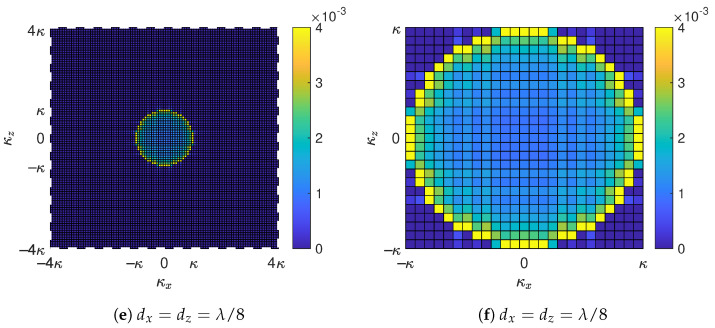
Overview (**a**,**c**,**e**) and zoom-in views (**b**,**d**,**f**) of the Fourier transform $G(\kappa_{x},\kappa_{z})$ in (23) of the truncated and discretized spatial correlation function $g(\Delta_{x},\Delta_{z})$ in (18). The colorbars represent the values of $G(\kappa_{x},\kappa_{z})$, and the white dashed frames in (**a**,**c**,**e**) outline the regions within which the wavenumbers are resolvable for the corresponding holographic RIS. Note that $\kappa_{x}$ and $\kappa_{z}$ are expressed in terms of the wavenumber κ which is directly labeled on the abscissa and ordinate in each subfigure.

To examine the impact of the spatial sampling interval on the power spectrum $G(\kappa_{x},\kappa_{z})$, we perform numerical simulations with a series of $\eta_{x}$ and $\eta_{z}$ values, and the corresponding results are shown in Figure 5. Figure 5a,c,e illustrate the power spectrum $G(\kappa_{x},\kappa_{z})$ evaluated at $\eta_{x}=\eta_{z}=1/2$, $\eta_{x}=\eta_{z}=1/4$, and $\eta_{x}=\eta_{z}=1/8$, respectively. For fair comparison, $\kappa_{x}$ and $\kappa_{z}$ range from −4κ to 4κ in all of the three subfigures, while the actual wavenumber regime that the corresponding holographic RIS can resolve is within the white dashed frame in each of the three subfigures. Figure 5b,d,f are the zoom-in views of Figure 5a,c,e, respectively, where $|\kappa_{x}|\leq \kappa$ and $|\kappa_{z}|\leq \kappa$. Since $b_{l,m}$ in (19) can be treated as an aperiodic discrete-space signal, its Fourier transform (i.e., spectrum in the wavenumber domain) $G(\kappa_{x},\kappa_{z})$ in (23) is periodic with periodicities of $\kappa_{x}/\eta_{x}$ and $\kappa_{z}/\eta_{z}$ along the *x*- and *z*-directions, respectively, as demonstrated in Figure 5a,c,e. Therefore, the spectrum in the wavenumber domain is actually superpositions of the original spectrum and its replicas, and the smaller the element spacing, the more serious the aliasing is. Due to the presence of spectrum leakage outside the region D(κ) in (27) as shown by Figure 4, the spectrum aliasing magnifies some of the spectrum values around the periphery of D(κ), and this magnification is more evident for smaller element spacing, as evidenced in Figure 5b,d,f. Recall that the eigenvalues of $R_{0}/N$ are asymptotically equally distributed with the corresponding power spectrum $G(\kappa_{x},\kappa_{z})$, hence the number of significant eigenvalues appears larger for smaller element spacing, which explains the observation highlighted by the dark circles in Figure 3. In other words, the number of eigenvalues lying in the far-field propagating wave region D(κ) in (27) actually does not change with the element spacing, the specious more dominant eigenvalues and hence higher spatial DoF for a smaller number of elements are contributed by evanescent waves outside the region D(κ).

Evanescent waves are usually localized in the reactive near field of an array. They contain the high-spatial-frequency components of an object, and normally do not contribute to the far-field channel capacity, but have a huge potential in near-field communication, sensing, and power transfer [53,54]. Moreover, diverse approaches have been put forward to convert evanescent waves to propagating waves that can be radiated to the far field, e.g., via simple metal strip gratings, metasurfaces, or randomly distributed metal wires [55,56,57]. Overall, holographic RISs, along with the associated propagating and evanescent waves, can find expansive potential applications in future communications, sensing, and related realms.

## 4. Eigenvalue Distributions with MC

In this section, we investigate eigenvalue distributions taking into account MC in holographic RISs.

### 4.1. Coupling Matrix

The impedance matrix Z of an antenna array can be expressed as

(28) $$Z=\left[\begin{array}{cccc} z_{A} & z_{12} & \cdots & z_{1N}\\ z_{21} & z_{A} & \cdots & z_{2N}\\ \vdots & \vdots & \ddots & \vdots \\ z_{N1} & z_{N2} & \cdots & z_{A} \end{array}\right]$$

with $z_{A}$ denoting the antenna impedance, and $z_{mn}$ the mutual impedance between the *m*-th and *n*-th elements. Given Z, the (unnormalized) coupling matrix of the array at the Tx side can be calculated based on circuit theory as [30,34]

(29) $$C_{T}^{\prime }=Z{\left(Z+z_{S}I_{N}\right)}^{-1}$$

where $z_{S}$ is the source impedance. If there is no MC between the Tx elements, then Z is diagonal with entries $z_{A}$, hence $C_{T}^{\prime }$ is diagonal as well with entries ${\left[C_{T}^{\prime }\right]}_{nn}=z_{A}/\left(z_{A}+z_{S}\right)$. Consequently, we normalize the coupling matrix by $z_{A}/\left(z_{A}+z_{S}\right)$ to obtain

(30) $$C_{T}=\left(1+z_{S}/z_{A}\right)Z{\left(Z+z_{S}I_{N}\right)}^{-1}.$$

On the other hand, the (unnormalized) coupling matrix of the array at the Rx side is given by [30,34]

(31) $$C_{R}^{\prime }=z_{L}I_{N}{\left(Z+z_{L}I_{N}\right)}^{-1}$$

where $z_{L}$ is the load or termination impedance. Analogous to the Tx side, if no MC exists between the Rx elements, then $C_{R}^{\prime }$ is diagonal with entries ${\left[C_{R}^{\prime }\right]}_{nn}=z_{L}/\left(z_{A}+z_{L}\right)$, such that the normalized coupling matrix is

(32) $$C_{R}=\left(z_{A}+z_{L}\right){\left(Z+z_{L}I_{N}\right)}^{-1}.$$

Given the coupling matrix, the effective spatial correlation matrix R for a holographic RIS can now be computed by applying the theoretical analysis in Section 2. The steps for deriving R are summarized below:

- Step 1: Calculate the conventional (i.e., MC-unaware) array response vector $a_{0}$ in (5) based on the geometry and element topology of the RIS;
- Step 2: Obtain the spatial scattering function s(ϕ,θ) in (3) based on theoretical analysis or measurements;
- Step 3: Calculate the spatial correlation matrix $R_{0}$ that excludes MC according to (6);
- Step 4: Obtain the impedance matrix Z in (28) for the elements in a holographic RIS based on theoretical analysis or measurements;
- Step 5: Calculate the coupling matrix at the Tx and Rx, $C_{T}$ and $C_{R}$, according to (30) and (32), respectively;
- Step 6: Calculate the effective spatial correlation matrix R according to (14) using $R_{0}$ from Step 3 and $C_{T}$ ($C_{R}$) from Step 5 for the Tx (Rx).

### 4.2. Inter-Element Correlation/Coupling Strength Indicator

To facilitate the quantitative description of the physical MC or correlation strength represented by a coupling or correlation matrix $Q\in C^{N\times N}$, we propound a new metric called *ICSI* defined as

(33) $$\begin{array}{cc} \mathrm{ICSI} & =\frac{1}{N}\sum_{n=1}^{N}\frac{\frac{1}{N-1}\sum_{m=1,m\neq n}^{N}|{\left[Q\right]}_{n,m}|}{\left|{\left[Q\right]}_{n,n}\right|}\\ \; & =\frac{1}{N(N-1)}\sum_{n=1}^{N}\sum_{m=1,m\neq n}^{N}\frac{\left|{\left[Q\right]}_{n,m}\right|}{\left|{\left[Q\right]}_{n,n}\right|} \end{array}$$

where $\frac{\left|{\left[Q\right]}_{n,m}\right|}{\left|{\left[Q\right]}_{n,n}\right|}$ represents the inter-element correlation/coupling strength between the *n*-th and *m*-th (m≠n) elements normalized by the self-correlation/coupling magnitude of the *n*-th element to ease the comparison between different matrices, then the normalized inter-element correlation/coupling magnitude is averaged over all pairs of distinct array elements to arrive at the mean normalized inter-element correlation/coupling strength, i.e., the ICSI. The value of ICSI lies between 0 and 1, which indicate no MC/correlation and maximum MC/correlation between different array elements, and roughly correspond to the highest and lowest level of orthogonality among the columns/rows of the correlation or coupling matrix Q, respectively. A large value of ICSI entails intense correlation/coupling between distinct array elements on average, which is likely to happen when the correlation/coupling between some pairs of elements is quite strong and/or a large amount of elements are mutually coupled or correlated, and this usually gives rise to unevenly distributed eigenvalues of the coupling/correlation matrix, as will be shown by the numerical results in the subsection below.

### 4.3. Numerical Simulations Including MC

**Figure 6:**
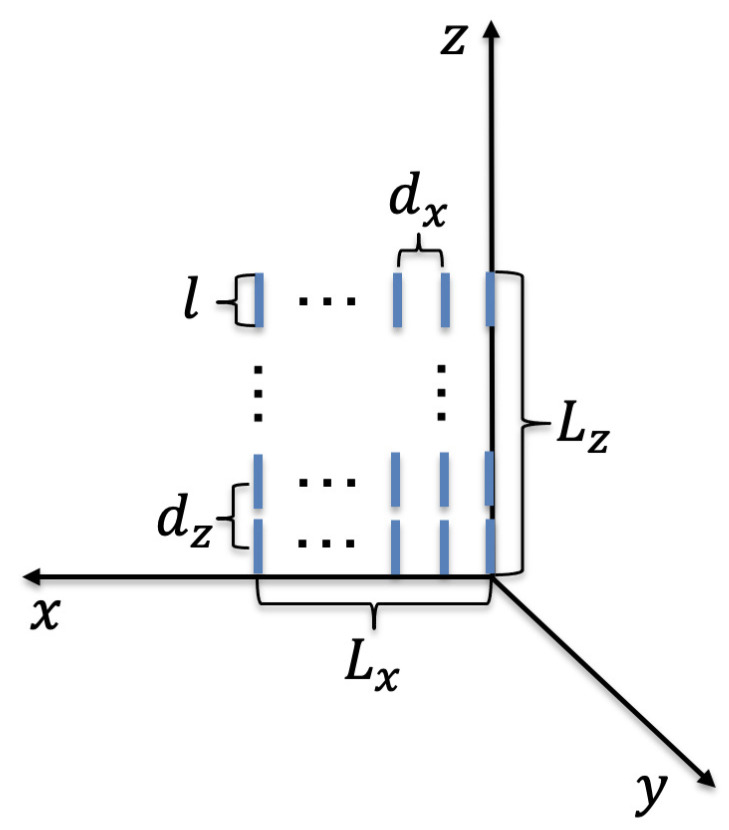
Dipole topology on the xoz plane with element spacing of $d_{x}$ and $d_{z}$, and array lengths of $L_{x}$ and $L_{z}$ on the *x*- and *z*-axes, respectively. Each dipole element is of length l=λ/2.

Due to the complexity of the MC phenomenon, it is impossible to show generic formulas or curves for the impedance matrix or coupling matrix that apply to all types of elements or arrays. For ease of exposition, we adopt the analytical equations for MC between identical small dipoles as an example. Although the expressions are derived based on two dipoles, they may be applied to any pair of elements in a linear or planar array with or without a ground plane [58]. When the elements are small and resonant, such as dipoles, the first-order result of MC is to alter the impedance of each of the array elements [27]. Thus, the coupling can be described in terms of mutual impedance between elements (when higher order effects can be neglected), which is also a common practice in plenty of previous research work (e.g., [35,37,39]). The mutual impedance and MC models in this paper are intended to be illustrative; for arrays composed of dipoles with other layouts or a different type of elements, it is necessary to employ more suitable mutual impedance/MC models, or to measure these parameters in the actual array, which is deferred to future work. In this paper, we employ the impedance matrix of half-wave dipole elements in a parallel-in-echelon configuration as an example, as illustrated in Figure 6, where $z_{A}\approx 73.1+j42.5\;\Omega$ [27,59]. In the simulations, $L_{x}=4\lambda$ and the number of half-wavelength dipoles along the *z*-axis is set to eight. The spacing between the upper end of a dipole and the lower end of the adjacent one above it along the *z*-axis is set to λ/50, which is negligibly small compared with λ so that $d_{z}\approx \lambda /2$ and $L_{z}\approx 4\lambda$.

**Figure 7:**
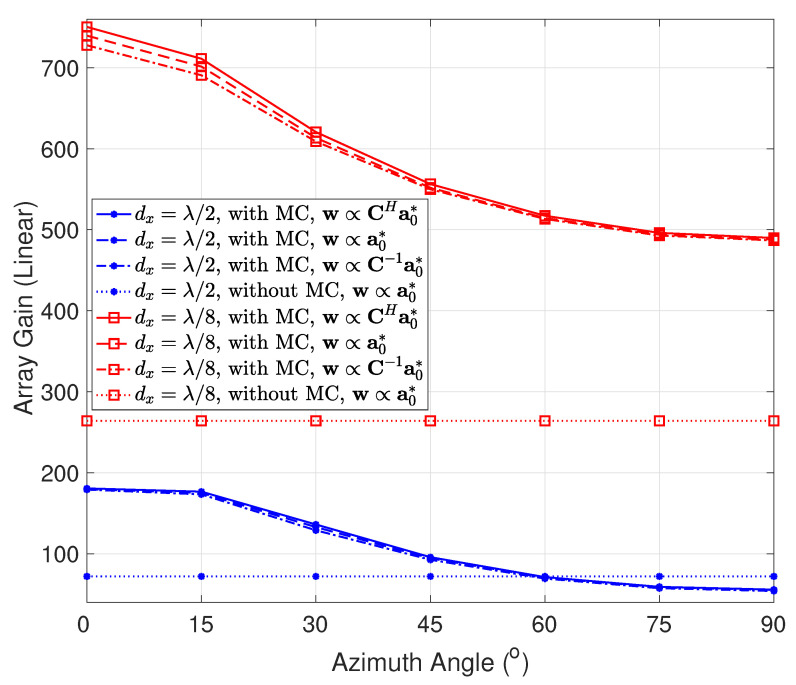
Transmit (Tx) array gain as a function of the azimuth angle for both without and with mutual coupling (MC) cases. The RIS size is 4λ×4λ, the vertical element spacing is about λ/2, and the zenith angle is set to $90^{\circ }$. $d_{x}$, w, C, and $a_{0}$ denote the element spacing along the *x*-axis, beamforming vector, coupling matrix in (30), and MC-unaware array response vector in (5), respectively.

As mentioned in Section 2.2, one of the distinctive features of a holographic RIS is that its array gain may be significantly larger than a conventional array; thus, let us first investigate the array gain of holographic RISs. As an instance, Figure 7 depicts the Tx array gain as a function of the azimuth angle ϕ (see Figure 1) for both without and with MC cases. The zenith angle θ (see Figure 1) is set to $90^{\circ }$. $d_{x}$, w, C, and $a_{0}$ denote the element spacing along the *x*-axis, beamforming vector, coupling matrix in (30), and MC-unaware array response vector in (5), respectively. The element spacing along the *x*-axis $d_{x}$ is set to λ/2 and λ/8, respectively, and four MC plus beamforming schemes are considered for each $d_{x}$. (Scheme 1): MC is included, and the proposed beamforming vector w in (12) is adopted. (Scheme 2): MC is included, and the ordinary conjugate beamforming $a_{0}^{*}$ (followed by power normalization) is employed. (Scheme 3): MC is included, and the existing beamforming method in [37] is utilized which maximizes the directivity of the RIS and is equivalent to $C^{-1}a_{0}^{*}$ (followed by power normalization). (Scheme 4): MC is excluded, and the optimal conjugate beamforming $a_{0}^{*}$ (followed by power normalization) is applied. The following remarks can be drawn from Figure 7: First, comparing the cases without and with MC, the array gain grows significantly in most cases when MC is included, even with the ordinary MC-unaware conjugate beamforming, except for some angles with the half-wavelength spacing. For example, the maximum achievable array gain using the proposed beamforming method is over 2.8 times that without MC for $d_{x}=\lambda /8$, and the gap is likely to expand as the RIS becomes denser, which is promising for SNR enhancement, coverage extension, and energy-efficient transmission for green communications. Second, when excluding MC, the array gain ratio between $d_{x}=\lambda /8$ and $d_{x}=\lambda /2$ equals the ratio of the corresponding number of elements (264/72≈3.7 herein) as expected, while this ratio reaches 8.8 when MC is included, indicating that the array gain incorporating MC increases more rapidly with the number of elements than the without MC scenario. Third, the proposed MC-aware beamforming approach outperforms its two counterparts including the one in [37]. The reason may lie in that the beamforming method in [37] is aimed to maximize the directivity of the RIS, which represents the radiation power for a certain pointing direction against that over all pointing directions, while the proposed beamforming vector maximizes the radiation power of an array against that of a single element, i.e., their optimization objects are slightly different. In general, the performance gaps among the three beamforming schemes increase with the element density and the proximity to the end-fire direction.

**Figure 8:**
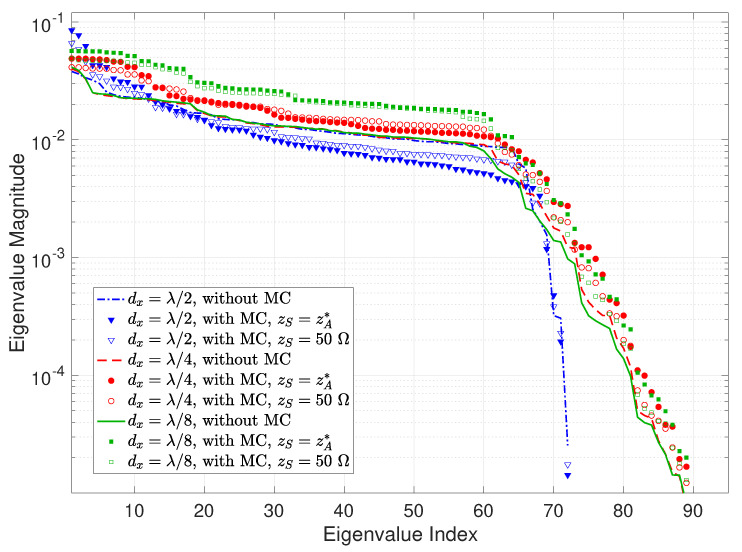
Eigenvalue magnitude versus eigenvalue index of the effective Tx spatial correlation matrix for horizontal element spacings of λ/2, λ/4, and λ/8 with a holographic RIS size of 4λ×4λ, for both without and with MC cases. $z_{S}$ denotes the source impedance. Note that both the eigenvalue index and eigenvalue magnitude are dimensionless.

Now, we inspect the eigenvalue behaviors of the effective spatial correlation matrix in (14) at the Tx and Rx, respectively, for different element intervals with a fixed holographic RIS size. Figure 8 depicts the eigenvalue magnitude of the effective Tx spatial correlation matrix for both without and with MC cases. Ideally, the source impedance would be the conjugate of the element impedance, i.e., $z_{S}=z_{A}^{*}$, but this is difficult to realize in practice. We thereby consider both perfect and imperfect impedance match by setting $z_{L}$ to $z_{A}^{*}$ and a common value of 50Ω to examine the influence of different impedance matching. Table 1 lists the associated ICSI calculated using (33), which includes another very large source impedance of 300Ω for comparison purposes. The following main remarks can be drawn from Figure 8 and Table 1:

- When excluding MC, the most prominent inflection point of the eigenvalues in Figure 8 shifts to the left, i.e., the number of dominant eigenvalues decreases, as the element spacing shrinks, which is consistent with the observations for the larger holographic RIS aperture in Figure 3 and is well explained by the analysis in Section 3.
- In most cases, MC increases the effective spatial correlation at Tx, as indicated by the more rapidly-decaying eigenvalues compared with the no MC case in Figure 8 as well as the ICSI values in Table 1, and also elevates the largest eigenvalues for all element spacings studied. Furthermore, the correlation enhancement by MC diminishes as the array becomes denser, and MC may even have a decorrelation effect for sufficiently dense RISs. The reason lies in the fact that the product term $Z{\left(Z+z_{S}I_{N}\right)}^{-1}$ in (30) approaches an identity matrix when $z_{S}\to 0$; meanwhile, for non-zero $z_{S}$, it becomes a banded symmetric block-Toeplitz matrix that is sparse with non-zero entries confined to a diagonal band, and the off-diagonal entries are significantly smaller than the diagonal ones. In addition, the sparsity becomes more pronounced as the number of elements increases. Consequently, the behavior of $C_{T}$ in (30) gradually resembles that of a diagonal matrix as the element spacing dwindles, such that the effective spatial correlation becomes weaker for smaller element spacing when including MC.
- Different source impedance values exert a noticeable effect on the eigenvalue structure. Specifically, the effective spatial correlation is enhanced more substantially by perfect impedance match $z_{S}=z_{A}^{*}$ in contrast to $z_{S}=50\;\Omega$, as demonstrated by the corresponding eigenvalue trends in Figure 8 and ICSI values in Table 1. This can be explained by similar reasons stated above: the properties of $C_{T}$ in (30) are farther apart from those of a diagonal matrix with a larger $z_{S}$ (i.e., 73.1−j42.5Ω, as opposed to 50Ω), thus higher correlation is induced. This is further verified by the ICSI for an even greater $z_{S}$ of 300Ω in Table 1.
- In general, the eigenvalue magnitude increases with the density of the holographic RIS, which is consistent with the array gain enhancement shown by Figure 7.

**Figure 9:**
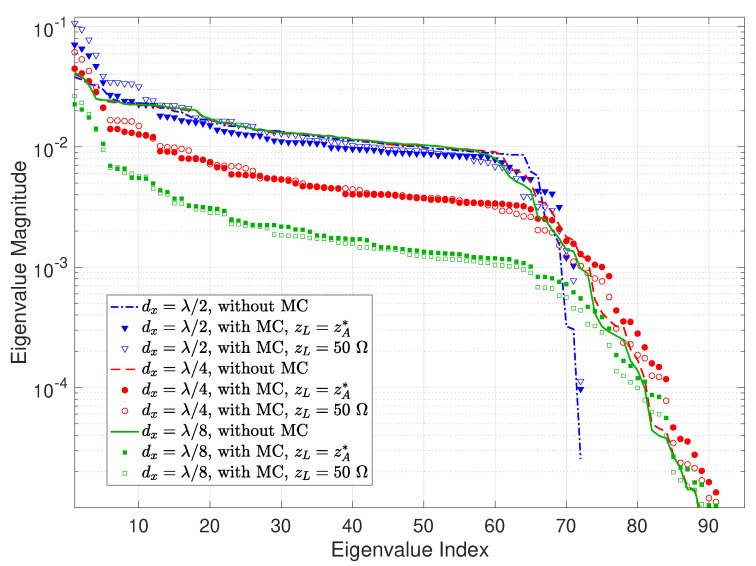
Eigenvalue magnitude versus eigenvalue index of the effective receive (Rx) spatial correlation matrix for horizontal element spacings of λ/2, λ/4, and λ/8 with a holographic RIS size of 4λ×4λ, for both without and with MC cases. $z_{L}$ denotes the load impedance. Note that both the eigenvalue index and eigenvalue magnitude are dimensionless.

The eigenvalue magnitude of the effective spatial correlation matrix at the Rx side is depicted in Figure 9 under the same simulation settings as in Figure 8, and the corresponding ICSI values are provided in Table 2. Some interesting phenomena are observed and described below.

- MC at the Rx reduces the magnitude of eigenvalues in most cases (except for the first few largest eigenvalues). Compared with the Tx coupling matrix $C_{T}$ in (30), the Rx coupling matrix $C_{R}$ in (32) is mainly different by a term $z_{A}Z^{-1}$, which causes the reduction of the eigenvalue magnitude after multiplying with the original spatial correlation matrix.
- As seen in Table 2, MC increases the effective spatial correlation at the Rx, which is similar to the Tx side. However, contrary to the observation for the Tx side, MC with a larger load impedance tends to have less correlation enhancement effect at Rx in general, as shown by Figure 9 and Table 2, since a larger load impedance renders the term $z_{L}I_{N}$ more dominant in ${\left(Z+z_{L}I_{N}\right)}^{-1}$ in (32) so that the coupling matrix is more like a diagonal matrix with smaller off-diagonal entries and hence weaker correlation.
- Rx MC increases the effective spatial DoF for element spacings less than half a wavelength, as implied by the most prominent inflection point of the eigenvalues which shifts to the right when considering MC. This is beneficial for spatial diversity and multistreaming.

**Figure 10:**
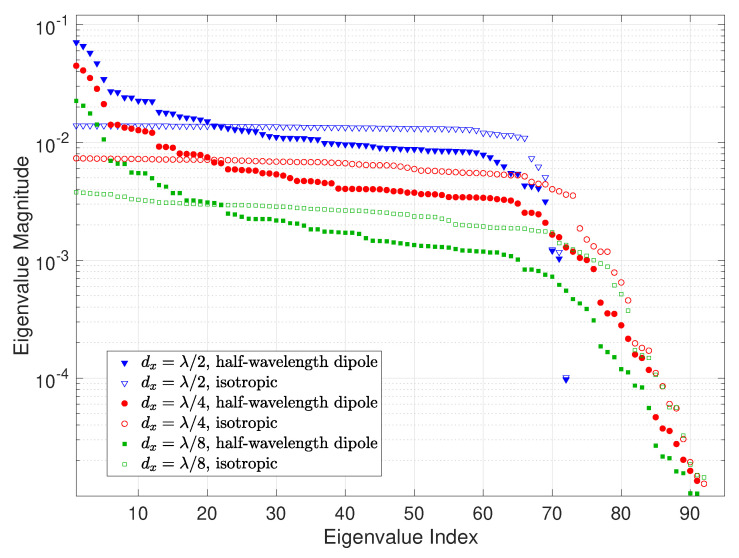
Eigenvalue magnitude versus eigenvalue index of the effective Rx spatial correlation matrix for half-wavelength dipoles and isotropic elements. The element spacing along the *x*-axis is set to λ/2, λ/4, and λ/8, respectively, with a holographic RIS size of 4λ×4λ, for both without and with MC cases. The load impedance $z_{L}=z_{A}^{*}$. Note that both the eigenvalue index and eigenvalue magnitude are dimensionless.

To analyze and compare the MC effects of different element types, we also look at the element with an isotropic radiation pattern which is commonly assumed in the existing work. The real part of the impedance matrix of isotropic elements is [47]

(34) $$\begin{array}{c} {\left[R\left\{Z_{\mathrm{iso}}\right\}\right]}_{n_{1},n_{2}}=r_{\mathrm{iso}}\sin c\left(\frac{2||d_{n_{1}}-d_{n_{2}}{\left|\right|}_{2}}{\lambda }\right),\;n_{1},n_{2}=1,\ldots ,N \end{array}$$

where $r_{\mathrm{iso}}$ denotes the radiation resistance of an isotropic element, and the definitions of sinc(x), $d_{n_{1}}$, and $d_{n_{2}}$ are aligned with those in (16). By applying appropriate matching techniques, the imaginary part of the impedance matrix can be removed while the real part remains [47], thus we only need to consider the real part. The Rx coupling matrix for isotropic elements is obtained by plugging (34) into (32) and setting $z_{L}=r_{\mathrm{iso}}$. Figure 10 displays the eigenvalue magnitude versus eigenvalue index of the effective Rx spatial correlation matrix for half-wavelength dipoles and isotropic elements, which shows that the effective spatial correlation for isotropic elements is obviously lower than that for half-wavelength dipoles, as manifest from the more evenly distributed eigenvalue magnitude for isotropic elements. This is because the MC is zero whenever the spacing between two isotropic elements is multiples of half a wavelength, as indicated by (34), while the MC is all non-zero for the dipoles herein, hence the overall MC is lower for isotropic elements.

## 5. Conclusions

In this paper, we have investigated the array response and small-scale spatial correlation for holographic RISs excluding and including MC. In-depth analysis is conducted on the asymptotic eigenvalue distribution and spatial DoF for the spatial correlation matrix under isotropic scattering, by linking the eigenvalues to the power spectrum of the spatial correlation function based on the BTTB matrix theory. It is demonstrated that the specious more dominant eigenvalues for fewer elements in holographic RISs are due to the spectrum aliasing in the wavenumber domain which enhances the near-field evanescent waves, thus the far-field spatial DoF actually does not increase. Furthermore, an MC-aware beamforming scheme is proposed which is aimed to maximize the array gain, and is shown to outperform existing methods. In addition, it is found that MC exerts discrepant effects on Tx and Rx modes: For the Tx, MC increases the eigenvalue magnitude and effective spatial correlation in most cases, while Rx MC often reduces the eigenvalue magnitude while increasing the spatial DoF, especially for dense RISs. The array gain and channel eigenvalue enhancement by Tx MC is beneficial to SNR elevation, coverage extension, and energy saving, and the growing spatial DoF caused by Rx MC indicates that efficient spatial multiplexing is possible even with compact arrays.

## Figures and Tables

**Table 1:** Inter-element correlation/coupling strength indicator (ICSI) in (33) for Tx spatial correlation matrix without and with MC under various element spacing.

ICSI in (33)	Tx Spatial Correlation Matrix without MC	Tx Spatial Correlation Matrix with MC, $z_{S}=z_{A}^{*}$	Tx Spatial Correlation Matrix with MC, $z_{S}=50\;\Omega$	Tx Spatial Correlation Matrix with MC, $z_{S}=300\;\Omega$
$d_{x}=\lambda /2$	0.0495	0.0927	0.0752	0.1500
$d_{x}=\lambda /4$	0.0646	0.0750	0.0665	0.0974
$d_{x}=\lambda /8$	0.0702	0.0705	0.0671	0.0851

**Table 2:** ICSI in (33) for Rx spatial correlation matrix without and with MC under various element spacing.

ICSI in (33)	Rx Spatial Correlation Matrix without MC	Rx Spatial Correlation Matrix with MC, $z_{L}=z_{A}^{*}$	Rx Spatial Correlation Matrix with MC, $z_{L}=50\;\Omega$	Rx Spatial Correlation Matrix with MC, $z_{L}=300\;\Omega$
$d_{x}=\lambda /2$	0.0495	0.0682	0.0787	0.0550
$d_{x}=\lambda /4$	0.0646	0.0867	0.1001	0.0698
$d_{x}=\lambda /8$	0.0702	0.1045	0.1213	0.0828

## Data Availability

Not applicable.
